# Quantifying late-stage host-seeking behaviour of *Anopheles gambiae* at the insecticidal net interface using a baited-box bioassay

**DOI:** 10.1186/s12936-020-03213-9

**Published:** 2020-04-07

**Authors:** Angela Hughes, Geraldine M. Foster, Amy Guy, Agnes Matope, Mayumi Abe, David Towers, Philip J. McCall

**Affiliations:** 1 Department of Vector Biology, Liverpool School of Tropical Medicine, Pembroke Place, Liverpool, L3 5QA UK; 2 Optical Engineering Group, School of Engineering, University of Warwick, Coventry, CV4 7AL UK

**Keywords:** Mosquito, Vector, Behaviour, *Anopheles*, ITN, Pyrethroid, Insecticide, Control, Bednet, ITN, Bioassay, Malaria

## Abstract

**Background:**

Insecticide-treated nets (ITNs) are losing efficacy against pyrethroid-resistant malaria vector populations throughout Africa. Safeguarding bed net efficacy, vital for effective malaria control, requires greater knowledge of mosquito–ITN interactions and how this impacts on the mosquito.

**Methods:**

A purpose-built benchtop apparatus with a closed 10 cm cubic chamber (the ‘Baited-box’) was used to video record behaviour of individual free-flying female *Anopheles gambiae* during approach and blood-feeding on a human hand through untreated nets and ITNs at close range. Time and duration of defined behavioural events, and knockdown and mortality at 1- and 24-h post-exposure respectively, were recorded for pyrethroid susceptible and resistant mosquitoes.

**Results:**

Using three human volunteers differing in relative attractiveness to mosquitoes, 328 mosquitoes were individually tested. There were no significant differences between response rates to ITNs and untreated nets (*P *> 0.1) or between resistant (Tiassalé) and susceptible (Kisumu) mosquito strains, at untreated nets (*P *= 0.39) or PermaNet 2.0 (*P *= 1). The sequence of behavioural events from host-seeking to completion of blood-feeding was consistent in all tests but duration and start time of events involving net contact were reduced or delayed respectively with ITNs. Blood-feeding durations at untreated nets (means from 4.25 to 8.47 min (95% confidence interval (CI) = 3.39–9.89) at 3 human volunteers) were reduced by 37–50% at PermaNet 2.0, in susceptible (mean 2.59–4.72 min, 95% CI = 1.54–5.5, *P *= < 0.05) and resistant (mean 4.20 min, 95% CI = 3.42–4.97, *P *= 0.01) strains. Total accumulated net contact was approximately 50% lower at PermaNet and Olyset ITNs (*P *< 0.0001) in susceptible (two of the three volunteers) and resistant mosquitoes. Times prior to first net contact were similar at untreated nets and ITNs (*P *> 0.2), and neither ITN type showed detectable spatial repellency. After initial contact, blood-feeding commenced later at Olyset (mean 2.76 min, 95% CI = 1.74–3.76, *P *= 0.0009) and PermaNet (mean 2.4 min, 95% CI = 1.52–3.33, *P *= 0.0058) than untreated netting (mean 0.68 min, 95% CI = 0.42–0.94).

**Conclusions:**

The baited box offers a simple method for detailed characterization of mosquito behavioural responses to insecticidal nets, for comparing entomological modes of action between nets and for defining the behavioural responses of particular mosquito strains or populations. The device has potential as a screening assay in the search for novel net treatments and for investigations into behavioural resistance mechanisms.

## Background

Insecticide-treated nets (ITNs) are a highly effective method for preventing malaria in Africa [1]. They are fundamental to the Global Malaria Action Plan (GMAP) and its aims of universal coverage for sustainable malaria reduction and eventual elimination in affected communities [2]. ITNs deliver doses of pyrethroid insecticide (standard ITNs) or pyrethroid plus a second active ingredient (next generation bed nets) when the mosquito makes contact with the net surface. However, widespread insecticide resistance in *Anopheles gambiae* sensu lato (*s.l.*) and *Anopheles funestus s.l.*, the two African malaria vector groups most effectively targeted by ITNs, is diminishing the effects of the insecticide dosage delivered such that standard ITNs have lost efficacy against resistant populations [3–8].

While the mechanisms of insecticide resistance in malaria vectors have been studied and characterized extensively at the molecular level, knowledge of behavioural changes associated with resistance is sparse, limited in part by the absence of suitable methods for exploring vector behaviour, as reiterated or clarified by many in the field [9–11]. In fact, describing the effects of mosquito-ITN contact still relies on a set of World Health Organization (WHO) recommended standard bioassays with protocols that record immediate knock down or mortality after 24–48 h, using non-blood fed adult female mosquitoes, exposed to the active ingredient under highly artificial and simplified conditions. Details of how mosquitoes interact with the active ingredients at a net’s surface have never been satisfactorily described and much remains unknown, e.g. what is the minimum duration of ITN contact necessary to deliver an effective dosage; do [some] nets have repellent properties that can prevent that threshold being reached; are there consequences of sub-threshold exposure that can affect vectorial capacity; how do all of these properties change as the net ages? Many of these knowledge gaps have been revisited since the DDT era [12–14], but satisfactory assays have yet to be developed. With the rising threat of pyrethroid resistance in Africa, recognition of the central role mosquito behaviour plays in determining the success or failure of ITN use has increased [9, 15].

Fundamental questions include clarification of the extent to which ITNs can or cannot repel host seeking mosquitoes. Laboratory studies have produced conflicting results, reporting that close to the ITN surface, repellency does [16–18] or does not [19, 20] occur. Clearly the net material, the insecticide used, the method of loading the net with active ingredient(s) and the age of the net are all important. Other variables may also influence these experiments: there is evidence that ITN irritancy and toxicity are reduced after blood-feeding [20, 21] and that sub-lethal exposure can affect responses to the host or ITN for up to 48 h after exposure [22]. Resistant mosquitoes have been reported to exhibit reduced, or total loss of, irritability/repellency in response to pyrethroids [16, 17, 23–25].

Successful development of novel ITNs and other tools will need a thorough understanding of the effects of insecticide treatments on these behaviours, and of how insecticide resistance status affects mosquito-net interactions. Appropriate bioassays that are based on natural behaviour will speed up the discovery process, reducing the time taken from product concept to wide-scale deployment. Previously, an infra-red tracking system was used to describe in detail the entomological mode of action of a standard pyrethroid-treated bed net [26]. That system provides considerable insight into behaviour during host-seeking at the entire human-baited ITN but cannot capture details of behaviour during the late stages of host location, as the mosquito arrives and lands on the bed net. This report describes the ‘Baited box test’, a benchtop bioassay system for characterizing the final sequence of behavioural events during landing and blood feeding on a human subject through insecticidal netting.

## Methods

### Baited box test apparatus and video

**Fig. 1:**
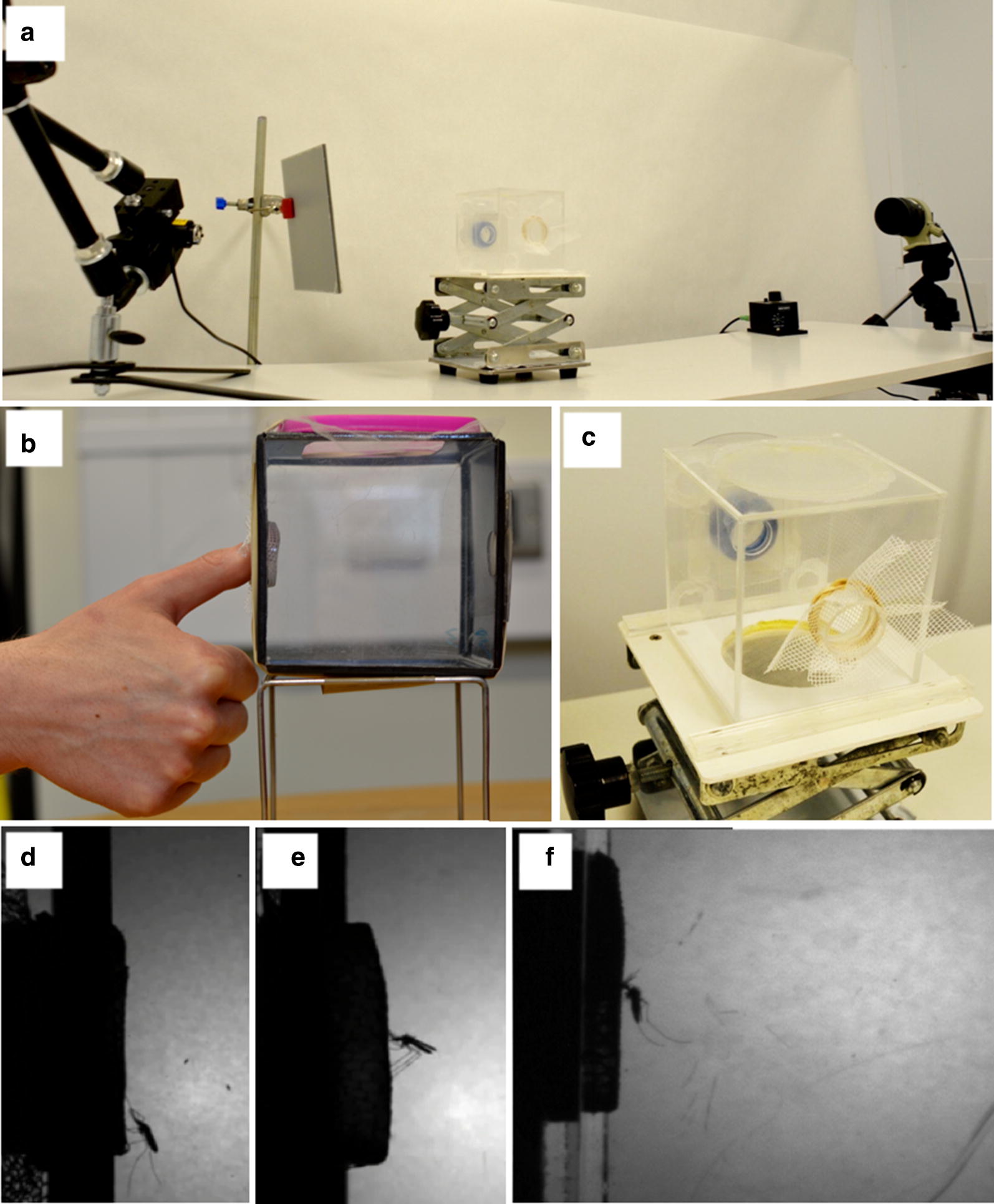
Baited box test apparatus and example images. **a** The complete bioassay and video system with (left to right): LED (not visible inside clamp); diffuser; test chamber on adjustable table; camera and Nikon lens. Test chamber **b** without entry port (early version) and, **c** with entry port (blue ring). Sample still images from test videos (60 mm lens), showing: **d** resting post-landing, **e** probing, and **f** blood feeding pre-defaecation

The test arena comprises a 10 × 10 × 10 cm clear plastic-walled test chamber with a 26 mm diameter port, through which a single mosquito is introduced (Fig. 1). On later designs, an entry tube was added to improve control of the mosquito’s release (Fig. 1b, c). The test netting is secured at a second 26 mm aperture on the opposite side, behind which the operator’s hand, forearm or thumb can be placed, to act as an attractants and blood source. Initial concern that behavioural effects of any vapour emitted by an entire side of the chamber (10 × 10 cm) would be amplified by a build-up of volatiles in the relatively small volume of the box, led to the use of this smaller test aperture. The 26 mm aperture is determined by the video camera field of view: fitted with a 60 mm lens, the camera captures the test net surface (Fig. 1d–f) while a 50 mm lens captures the entire box interior. With the 60 mm lens, all activity at the test net surface is captured in sufficient detail to observe the defined behavioural events (Fig. 1d–f). In tests reported here, the operator’s thumb was placed against the 26 mm test net aperture. All assays were performed in the dark.

The arena is illuminated by an infra-red light-emitting diode (850 nm, M850L2: Thorlabs, UK), positioned approximately 60 cm from the test chamber centre behind a diffuser placed 15–40 cm from the test chamber (Fig. 1a).

Mosquito behavioural responses to test netting are captured using a Dalsa Falcon 1.4Mp 100HG Camlink (Stemmer-imaging.com) camera fitted with a Nikon AF Micro-Nikkor 60 mm F2.8D lens, set at f#11 and positioned 30–60 cm from the test chamber centre (Fig. 1d). Images are recorded at 30 frames per second (fps) using commercial digital video recording software (either STEMMER CamExpert (CMV Movie Interactive 2) or StreamPix v.5, Norpix, Canada). Data are stored on external hard drives (e.g. Seagate 4 TB Backup plus, Amazon, UK). Video recordings are processed manually in real-time playback, with slow motion analysis as necessary.

### Mosquito behaviours measured

Defining the behaviours of interest for this study was directed by the need to explore events likely to be influenced or altered by insecticide residues on bed nets, within the confines of the tracking system’s capacity to record detail. Mosquito behaviours that were considered detectable and distinguishable by multiple observers during approach, landing and blood feeding were codified using an ethogram (Table 1). After reviewing the recorded videos, these nine events were combined for analyses into biologically relevant ‘Activities’ as shown in Table 1:

1. Response Lag Time—delay between test start and mosquito’s first entry into the field of view;
2. Time to first net contact;
3. Time until feeding—time from first net contact until probing stops;
4. Readiness to land—time between first contact with the net and cessation of flight;
5. Duration of blood meal—time from proboscis insertion into skin until withdrawal
6. Resting post feed—duration of net contact post blood meal
7. Total net contact—total accumulated net contact (coloured bars with black borders on schematic).

**Table 1:**
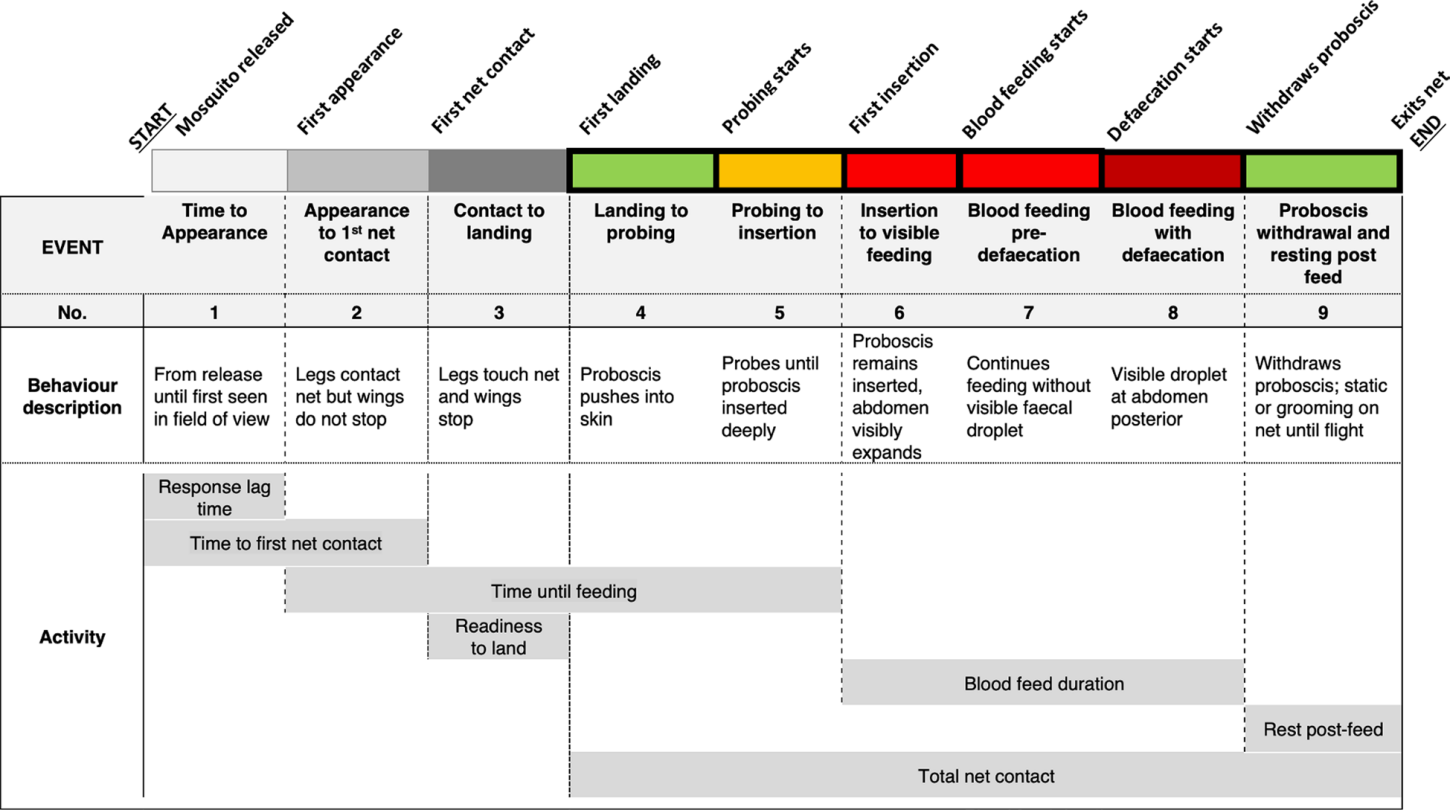
Ethogram of the behavioural events distinguishable during feeding on human bait through insecticide-treated netting

**Fig. 2:**
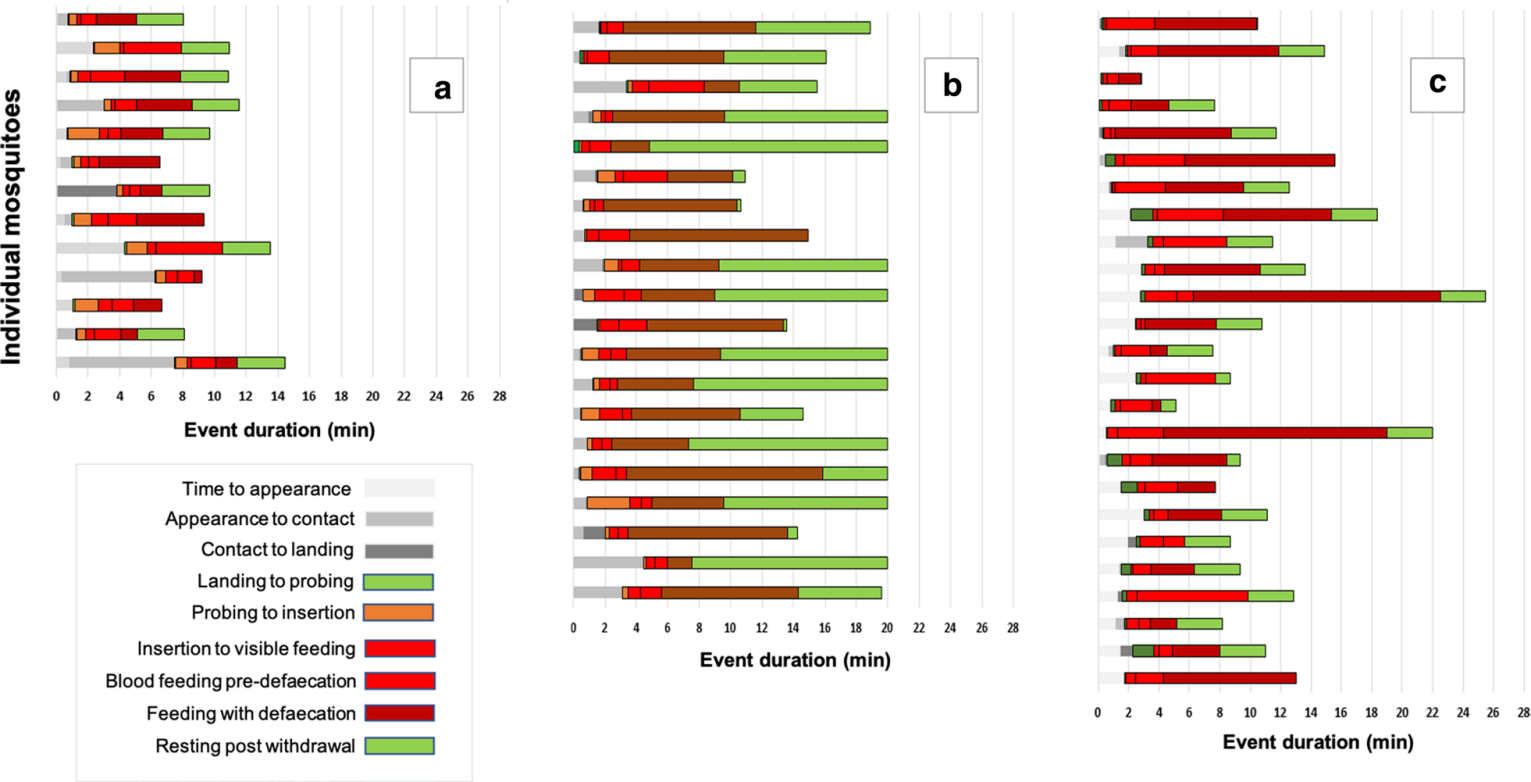
Activity of adult female *Anopheles gambiae s.s.* at untreated netting. The three Baited Box studies are shown separately, with behavioural events as defined in the ethogram in Table 1. Each line represents the responses and complete activity of a single mosquito, with duration of each behavioural event measured on the X-axis (0–28 min). On each chronogram, the initial grey-scale borderless sections represent activity prior to initial landing on the netting. The orange red and green segments with black borders are events that include contact with the test netting. In Study 1 (**a**), all events from initial net contact to proboscis insertion (Events 4 and 5) were combined into a single event. Throughout the test, mosquitoes were allowed to exit the release point and approach the operator thumb, probe, blood feed and depart from the test net without interference. In Studies 1 (**a**) and 2 (**b**), post-feeding resting on the net was limited to 3 min whereas in Study 3 (**c**), post-feeding resting could continue up to a total test duration of 20 min

**Fig. 3:**
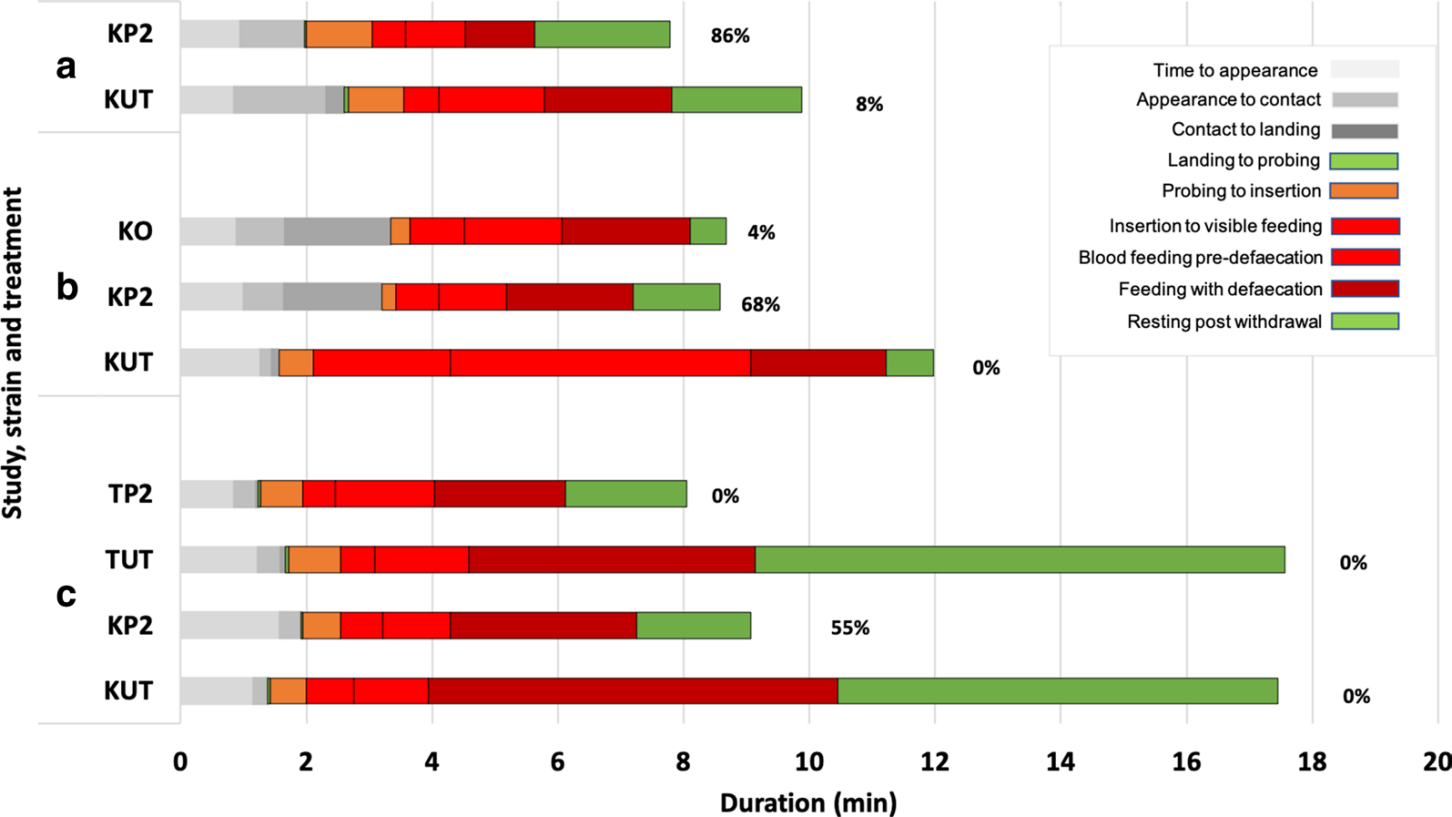
Blood feeding behaviour of insecticide susceptible and insecticide resistant *Anopheles gambiae* at pyrethroid treated netting Stacked barcharts showing responses of pyrethroid susceptible Kisumu (K) and Tiassale (T) strains in Baited Box tests of untreated (UT), PermaNet (P2) or Olyset (O) netting in Study 1 (**a**), Study 2 (**b**) and Study 3 (**c**). Each stacked bar chart represents the mean durations of each coded behavioural event in each test (Study 1: n = 13 for untreated net and 7 for PermaNet, Study 2: n = 25 mosquitoes per group, Study 3: n = 20 mosquitoes per group). The percentage mortality at 24 h post testing for each treatment group is shown at the end of each bar. In Studies 1 and 2, post-feeding resting on the net was limited to 3 min whereas in Study 3, post-feeding resting could continue up to a total test duration of 20 min

The table shows the start and endpoint of each event, and how they were grouped for each ‘Activity’. Note that although events are presented in a sequence typifying an uninterrupted blood meal, individual mosquitoes could exit at any point and return to an earlier behaviour to resume the sequence or terminate. 5*: events 4 and 5 were combined in Study 2. The colours in the horizontal bar correspond with those in Figs. 2 and 3: grey sections with no border show activity prior to first net contact; coloured sections with black borders represent behaviours involving net contact. The labels are also shown as subtitles marking the event boundaries in the accompanying video

### Study design

In these studies, the behavioural responses of *An. gambiae s.l.* female mosquitoes to treated and untreated netting were compared using three operators (AG, MA and AH), who also acted as hosts and hence were assumed to differ in their inherent attractiveness to mosquitoes and in their ability to manipulate them safely during transfer before and after bioassays. There were additional slight but notable differences between the three studies.

- *Study 1:* operator AG characterized blood-feeding behaviour of the insecticide-susceptible (IS) Kisumu strain at untreated net and one type of ITN.
- *Study 2*: MA characterized blood-feeding behaviour of the insecticide-susceptible (IS) Kisumu strain at three different bed net interfaces: two ITNs and untreated control.
- *Study 3* AH characterized blood-feeding behaviour of the Kisumu strain (IS) and the pyrethroid-resistant (IR) Tiassalé strain at untreated netting and one type of ITN.

In Studies 1 and 2 knock down at 60 min and mortality at 24 h was recorded; only 24 h mortality was recorded in Study 3.

### Mosquitoes

Unfed female adults (aged 3–5 days post-eclosion) of either the IS Kisumu or IR Tiassale strains of *An. gambiae s.l.* were used in all experiments. The Kisumu colony of *An. gambiae* sensu stricto (*s.s.*), originating from Kenya, was colonized in 1953 [27]. It has been maintained at the Liverpool School of Tropical Medicine (LSTM) since 1975. The Tiassalé strain was colonized from Southern Côte d’Ivoire in 2013 and maintained at LSTM under six-monthly selection pressure with deltamethrin. This strain, which contains both *An. gambiae* and *Anopheles coluzzii,* is resistant to pyrethroids and DDT [28]. It has a high frequency of 1014F *kdr* and ace-1 mutations and expresses elevated levels of key P450s known to metabolize pyrethroids.

Mosquitoes were starved of sugared water for at least 5 h prior to testing and were transferred to the experimental room at least 1 h prior to experiments. Lighting was dimmed and assays were performed after the first hour of the scotophase. Inactive mosquitoes that were not seen in the chamber within 5 min (3 min in Study 1) or active mosquitoes that had not begun probing within 10 min were discarded as non-responders.

Bioassays were run until 25 mosquitoes per treatment group had responded in Studies 1 and 2, and 20 per treatment group in Study 3. All experiments were performed in a climate-controlled insectary (27 ± 2 °C, 80 ± 8% RH) at the Liverpool School of Tropical Medicine, UK.

### Insecticidal netting

Netting from two commercially available ITN brands readily available and used widely across Africa were tested.

1. PermaNet^®^ 2.0 (deltamethrin 55 mg/m^2^; 75 denier polyester; Vestergaard, Lausanne, Switzerland), hereafter PermaNet.
2. Olyset^®^ Net (permethrin 20 g/kg ± 3 g/kg; > 150 denier polyethylene; Sumitomo Chemical Co. Ltd., Tokyo, Japan), hereafter referred to as Olyset.

Untreated control nets were cut from polyester netting with mesh size similar to PermaNet, obtained locally in the UK and confirmed as non-insecticidal by WHO cone tests. All new ITNs were removed from packaging and hung indoors for at least 1 week before testing, to minimize any confounding risk caused by volatile contaminant odours deriving from the manufacturing or packaging processes.

### Data analysis

The Fisher’s exact test and Baptista-Pike method (for odds ratio) was used to compare the mosquito response rates. Behavioural events were analysed separately and in combination; comparisons between event durations were performed using ANOVA (Tukey’s multiple comparisons test). In Study 1, the number of replicates was too low to analyse by ANOVA, in these cases, a non-parametric unpaired T-Test (Mann–Whitney) was used. All above analyses were carried out using GraphPad Prism v8.1.2. (GraphPad Software Inc, CA, USA). Stacked bar charts of coded behavioural events were created using GraphPad Prism v7.03.

### Ethical considerations

The Liverpool School of Tropical Medicine Research Ethics Committee approved the study (‘Behaviour of African malaria vectors’: Permit number 12.13, issued 24th May 2012).

## Results

### Mosquito response rates and test duration

A total of 328 individual mosquitoes were tested of which 175 (53%) responded (Table 2). The total numbers of mosquitoes exposed were dictated by the propensity of mosquitoes to enter the video testing arena and successfully blood feed. Conditions and endpoints were slightly different in each trial but generally 30 min was required to complete one bioassay, including time for setting up and post-test cleaning. The most rapidly responding mosquitoes exited the entrance tube, completed feeding and rested within 20 min or less. The low response rate in Study 1 prevented the target of 25 mosquitoes per treatment group being reached in the available time. Response rates were significantly lower in Study 1/operator 1 compared with Study 2/operator 2 (Odds Ratio (OR) = 0.112, 95% CI = 0.04–0.32*, n *= 81*, P *= < 0.0001) and Study 3/operator 3 (OR = 0.09, 95% CI = 0.03–0.27 *n *= 79, *P *= < 0.0001). Response rates for studies 2 and 3 did not differ (OR = 1.3, 95% CI = 0.44–4.81, *n *= 64*, P *= 0.77).

**Table 2:** Mosquito response rates in three studies with three different operators/hosts

	Response rate (%)			
	No mosquitoes responding/no. tested			
Study	Study 1	Study 2	Study 3	
Operator	AG	MA	AH	
Mosquito strain	Kisumu	Kisumu	Kisumu	Tiassalé
Treatment				
Untreated	27% (13/48)	76% (25/33)	77% (20/26)	67% (20/30)
PermaNet 2	18% (7/38)	58% (25/43)	61% (20/33)	61% (20/33)
Olyset		57% (25/44)		
Total no. tested	86	120	59	63

Within each study, there were no significant differences in responses to Untreated nets and PermaNet 2.0 or Olyset ITNs: Study 1: *X*^2^ (1, *n *= 86) = 0.89, p = 0.35, OR = 0.61, 95% CI = 0.22–1.72); Study 2: (1, *n *= 75) = 2.57, p = 0.11, OR = 0.77, 95% CI = 0.37–1.58); Study 3 (Kisumu) (1, *n *= 59) = 1.77, p = 0.18, OR = 0.79, 95% CI = 0.35–1.76) and Study 3 (Tiassalé) (1, *n *= 63) = 0.25, p = 0.617, OR = 0.91, 95% CI = 0.41–2.01)

Response rates to ITNs were not significantly different to untreated nets. In all tests, there was no indication that the insecticide treated net reduced a mosquito’s readiness to respond to the host, prior to net contact (Study 1: OR = 1.65, 95% CI = 0.56–4.25, *n *= 86, *P *= 0.44; Study 2: OR = 2.25, 95% CI = 0.84–6.36*, n *= 75, *P *= 0.15; Study 3 (Kisumu) OR = 2.17, 95% CI = 0.70–7.37, *n *= 59, *P *= 0.26 and Study 3 (Tiassalé) OR = 1.3, 95% CI = 0.49–3.57, *n *= 63, *P *= 0.80). Response rates of mosquitoes from a resistant strain (Tiassalé) did not differ from the susceptible strain (Kisumu) during exposure to untreated nets (OR = 1.6, 95% CI = 0.48–5.7, *n *= 56, *P *= 0.39) or PermaNet ITNs (OR = 1, 95% CI = 0.35–2.8, *n *= 66, *P *=>0.99).

### Blood-feeding behaviour of pyrethroid susceptible *Anopheles gambiae* at untreated netting

The sequence of behaviours observed between a mosquito’s arrival at the bed net surface, through probing and blood feeding until its departure from the net, are presented in Fig. 2, Table 3 and in Additional file 1: supplementary video. This sequence of events was similar in all studies, but the duration of certain key events was highly variable.

**Table 3:** Duration and percentage proportion of selected events for the IS Kisumu strain of *Anopheles gambiae* when exposed to PermaNet and Olyset in the Baited box test

Event^a^	1	3	2, 3, 4, 5	6,7,8	4–9	9
Activity^a^	Response lag time	Readiness to land	Appearance to blood feed start	Blood feed duration	Total net contact duration	Resting post feed
Net	Mean duration (minutes), SD, range, proportion of total assay length					
Study 1						
UT	0.85 1.19 0–4.25 8%	0.29 1.03 0–3.73 3%	2.71 2.14 1.27–8.28 27%	4.25 1.42 2.27–7.05 45%	7.58 2.0 2.97–9.98 78%	2.08 1.44 0–3 20%
P2	0.94 0.79 0.13–2.05 14%	0.01 0.012 0–0.3 0.11%	2.11 0.93 1.18–3.47 28%	2.59 1.13 1.57–4.58, 34%	5.82 2.3 2.4–7.95 73%	2.14 1.46 0–3 24%
Study 2						
UT	1.26 0.94 0.01–1.16 11%	0.11 0.18 0–0.77 1%	0.68 0.63 0.09–2.5 6%	7.50 4.66 2.48–19.4 65%	10.04 4.80 2.65–22.7 87%	2.16 1.28 0–3 19%
P2	0.99 1.04 0.05–2.7 12%	1.56 2.17 0.02–8.12 18%	2.43 2.18 0.2–8.25 28%	3.77 1.64 1.1–7.08 44%	5.38 1.93 1.9–9.2, 63%	1.38 1.14 0–3 16%
O	0.89 1.10 0.02–0.35 10%	1.69 2.10 0.02–8.07 20%	2.76 2.44 0.3–8.5 32%	4.45 1.61 1.7–7.5 51%	5.33 1.98 2.2–9.7 61%	0.57 0.93 0–3 7%
Study 3						
UT	1.15 1.20 0.65–1.75 7%	0.01 0.02 0–0.017 < 1.0%	0.85 0.68 0.15–2.78 5%	8.47 1.67 7.04–9.87 49%	16.10 3.43 14.47–17.68 92%	7.17 3.80 4.67–9.32 40%
P2	1.57 1.41 0.91–2.23 17%	0.01 0.02 0–0.05 < 1.0%	0.98 0.78 0.17–2.6 11%	4.72 1.67 3.93–5.49 52%	7.17 3.79 5.38–8.94 79%	1.81 3.48 0.17–3.44 20%

The proportion of recorded time spent on each event is shown as a percentage of total assay length. Untreated net data included for comparison purposes. (^a^see Table 2)

Prior to blood-feeding: lag times ranged from 0 to 4.25 min, times from appearance to first net contact from 0 to 7.47 min, and first net contact to initiation of probing 0.09–8.28 min. The three events comprising blood feeding duration ranged from 2.27 to 19.4 min. Post-feeding, mosquitoes remained on the net surface, either resting or grooming, for between 0 and 9.32 min. Total net contact times were recorded at a range of between 2.65 and 22.7 min.

There were significant differences between the three studies in some key events (Fig. 2, Table 3). Blood-feeding duration was significantly longer in Study 3 (mean 8.47 min, 95% CI = 7.05–9.88) than in Studies 1 (mean 4.25 min, 95% CI 3.39–5.11, *P *= 0.005) and 2 (mean 7.5 min, 95% CI = 5.57–9.42, *P *= 0.029). Time until blood feeding was significantly longer in Study 1 (mean 2.71 min, 95% CI = 1.42–3.99) compared to Study 2 (mean 0.68 min, 95% CI = 0.42–0.94, *P *= < 0.0001) and Study 3 (mean 0.85 min, 95% CI = 0.53–1.16, *P *= < 0.0001).

Following untreated net exposure, knock down at 60 min and/or mortality at 24 h was < 10% in all studies.

### Blood-feeding behaviour of pyrethroid susceptible *An. gambiae* at an insecticide-treated net

The basic sequence of events was not different to an untreated net (Fig. 3; Table 3). However, the duration of blood feeding was reduced by 37–50% at ITNs, significantly shorter than on untreated netting (PermaNet: Study 1, mean 2.59 min, 95% CI = 1.54–3.63, *P *= 0.01; Study 2, mean 3.77 min, 95% CI = 3.10–4.45, *P *= 0.0001; Study 3, mean 4.72 min, 95% CI = 7.05–9.89*, P *= < 0.0001; Olyset (Study 2 only): mean 4.45 min, 95% CI = 3.8–5.12, *P *= 0.0017; PermaNet vs Olyset (*P *= 0.699).

Total net contact duration was also reduced on ITNs compared with untreated netting in Study 2 (PermaNet, mean 5.38 min, 95% CI = 4.6–6.17, *P *= < 0.0001 and Olyset, mean 5.33 min, 95% CI = 4.52–6.2, *P *= < 0.0001 versus untreated net, mean 10.04 min, 95% CI = 8.05–12.02) and Study 3 (PermaNet, mean 7.17 min, 95% CI = 5.4–8.9, *P *= < 0.0001 versus untreated net, mean 16.10 min, 95% CI = 14.5–17.7).

Time to first net contact (events 1 and 2) is the period when volatile chemicals from the net would exert an influence on behaviour (i.e. true repellency rather than excito-repellency). With no significant difference in the duration of this activity between untreated nets and PermaNets (*P *> 0.3) or Olysets (*P *= 0.21) or between both ITNs (*P *= 0.93), these results indicate that neither ITN has spatial repellent properties.

However, after initial contact, there was a significant increase in the time from appearance to commencement of blood feeding (willingness to feed) with Olyset (mean 2.76 min, 95% CI = 1.7–3.8, *P *= 0.0009) and PermaNet (mean 2.4 min, 95% CI = 1.52–3.33, *P *= 0.0058) compared to untreated netting (mean 0.68 min, 95% CI = 0.42–0.95). There was also a difference in the time from first net contact to landing (readiness to land) on ITNs in Study 2 (PermaNet, mean 1.56 min, 95% CI = 0.67–2.5, *P *= 0.012; Olyset, mean 1.69 min, (95% CI = 0.84–2.56, *P *= 0.006) compared to untreated netting (mean 0.11 min, 95% CI = 0.04–0.19).

In Study 1 (PermaNet), both knockdown and 24 h mortality were 86%; in Study 2, knockdown at 60 min was 96% (24/25) and 24% (6/25), and mortality at 24 h was 68% (17/25) and 4% (1/25) for PermaNet and Olyset, respectively. In Study 3 (PermaNet), at 24 h post-exposure, mortality was 55%.

### Blood feeding behaviour of pyrethroid resistant *An. gambiae* at an insecticide-treated net

The behaviour of pyrethroid resistant mosquitoes did not differ from that of susceptible strains and the effects of ITNs on the behaviour of both strains were similar (Fig. 3). When resistant mosquitoes fed through PermaNet ITNs, the duration of blood feeding (mean 4.20 min, 95% CI = 3.42–5.0), post feed resting (tolerance of irritation (mean 1.93 min, 95% CI = 0.44–3.41) and total accumulated time in contact with the net (mean 6.87 min, 95% CI = 4.96-8.77) were significantly lower (*P *= < 0.001) than at untreated controls (blood feeding: mean 6.60 min, 95% CI = 5.28–7.91; resting: mean 8.43 min, 95% CI = 6.0710.78 and total net contact: mean 16.10 min, 95% CI = 14.49–17.7). Mortality of the IR strain was zero at 24 h post exposure.

## Discussion

This study describes, at an exceptional level of detail, the behaviour of host-seeking *An. gambiae* during the critical final stages of arrival, landing and blood-feeding through untreated and insecticide-treated bed nets. The results reveal a highly-conserved behaviour. Human attractiveness to mosquitoes varies between individuals and could account for the differences in response rates and at least some of the variation in duration of mosquito behaviours between the three operators. However, regardless of operator, net treatment or mosquito strain, the behavioural responses recorded with this system were remarkably consistent across all tests.

At untreated nets, behaviour of all mosquitoes, whether susceptible or resistant to pyrethroids, followed an ordered sequence of events (Table 1), though there was considerable variation in the duration of each stage in the sequence. At insecticide-treated nets (PermaNet and Olyset ITNs), the sequence of events was the same as at untreated nets, but the durations of blood-feeding and resting post-feeding and the total time accrued in contact with the net, were significantly reduced. The time from appearance to commencement of blood feeding was slightly higher than at untreated nets.

The Baited Box Test partners the room-scale flight tracking system [26, 29] to provide details of the closing stages of mosquito-net interaction that are not captured in the larger-scale assay. Applied to the PermaNet ITN, findings from this and the room-scale test [26] are compatible, defining it as an ITN with negligible repellency. Even at very close range, the mosquito’s behaviour prior to contact is virtually indistinguishable to that seen at untreated nets. Post-contact, the PermaNet’s irritant properties reduce net contact, before, during and after blood-feeding. Based on the limited number of tests performed to date, the responses to an Olyset are not dissimilar, but tracking flight data and tests with additional mosquito strains are needed before drawing a conclusion. These results also serve to illustrate the importance of maintaining a balance between the toxic/lethal and repellent/irritant properties of an ITN to ensure a lethal dose is delivered before a mosquito leaves the net.

Previous studies [30] and [31] have reported reductions in feeding rates of resistant *Anopheles spp.* on pyrethroid-treated nets, with Glunt et al. [30] reporting up to 60% reduction when mosquitoes were offered a blood meal immediately after ITN exposure. Hauser et al. [20] reported that pyrethroid susceptible *An. gambiae* mosquitoes commenced blood feeding later (53.5 s vs. 35 s after start of test) and spent less time feeding (40 s vs. 175 s) through an Olyset Plus insecticidal net than through untreated netting, respectively. The baited box setup permits measurement in finer detail than was possible in these studies, e.g. one can distinguish between behaviours prior to net contact (true volatile repellency) and those that occurred after net contact (contact irritation/excito-repellency), a detail that eluded Hauser et al. [20] who could not conclude whether or not their Olyset Plus exhibited spatial repellency.

Experimental results showed that an average of 55% of blood-fed susceptible individuals survived 24 h after blood feeding through a PermaNet. Almost certainly the result of reduced net contact, this phenomenon was first reported over 30 years ago by Hossain and Curtis [32] who reported 40% survival in *An. gambiae* after feeding through permethrin-dipped nets. Others found evidence for the blood meal’s protective effect in resistant strains of *An. funestus* and *Anopheles arabiensis* [21, 33, 34] and in a wild population in Burkina Faso [35]. Recently, Hauser et al. reported 85% survival rates in susceptible *An. gambiae,* 24 h after exposure [20].

The Baited box test can contribute to the search for next-generation insecticidal netting in the laboratory as a screening assay for new insecticides, to evaluate performance against, or to monitor resistance in, a vector population and as a bioassay to measure durability of aged nets. The current testing pipeline for new insecticidal vector control products typically follows the WHO Pesticide Evaluation Scheme (WHOPES), which involves phase I laboratory testing, phase II small scale field trials and phase III large scale field trials. However, the assays currently used in phase I evaluations, the WHO cone, tube and tunnel tests, are poorly linked to most products’ actual modes of action in field settings, as they record knockdown and mortality after brief, but forced, exposure providing no data on duration of contact with the insecticide, a key element of any net’s power to deliver a lethal dose. In contrast, the Baited-box test measures behaviour of free-flying mosquitoes attempting to feed on a human host behind a bed net, thereby quantifying behaviour in a far more field-relevant setup than the existing tests.

## Conclusion

The Baited Box test was developed to accelerate the development and delivery of effective vector control products to communities at risk of malaria, by providing the evidence required to select the most appropriate ITN to target that vector population and for routine use thereafter in monitoring it for insecticide resistance. In today’s ‘post-pyrethroid era’, assessment of efficacy in the field must capture far more than immediate knockdown and mortality effects, as was done for pyrethroid-only nets. The results presented in this report demonstrate the Baited-box’s potential as a screening assay for that purpose. The outputs from studies where the Baited box was used to evaluate impacts of next-generation ITNs in the field and laboratory will be available to report in the immediate future.

## Supplementary information

**Additional file 1.** Baited Box Assay video. Complete video recording of a Baited box assay. A single female *Anopheles gambiae* (pyrethroid resistant Tiassale strain) feeding on a human thumb through insecticide treated netting (PermaNet 2.0). The definitions of each behaviour event, activity and the colour scheme of the subtitles in the video correspond with those in Table 1. The video is the complete unedited recording and is reproduced at natural speed. The key timepoints that mark the divisions between each event are labelled in the video as subtitles, in the following sequence: (1) START/Mosquito released 00.00 (2) First appearance in field of view 00.26 (3) First net contact 01.11 (4) First landing on net 01.11 (5) Probing starts 01.12 (6) First insertion of proboscis 01.41 (7) Blood feeding starts 01.57 (8) Defaecation starts 03.45 (9) Withdraws proboscis 05.18 (10) Exits the net 05.23/END.

## Data Availability

The datasets used in this study are available from the corresponding author on reasonable request.
